# One-Year Outcome of Drug-Coated Balloon vs. Drug-Eluting Stent in Patients Undergoing Initial Percutaneous Coronary Intervention (PCI) for De Novo Lesion

**DOI:** 10.7759/cureus.56346

**Published:** 2024-03-17

**Authors:** Jun Goto, Takeshi Niizeki, Tadateru Iwayama, Toshiki Sasaki, Masafumi Watanabe

**Affiliations:** 1 Department of Cardiology, Okitama Public General Hospital, Yamagata, JPN; 2 Department of Cardiology, Pulmonology, and Nephrology, Yamagata University School of Medicine, Yamagata, JPN

**Keywords:** debulking device, percutaneous coronary intervention, target lesion failure, drug-eluting stents, drug-coated balloon

## Abstract

Background: Drug-eluting stents (DES) are the major treatment option in percutaneous coronary intervention (PCI). Recently, drug-coated balloon (DCB) utilization has been increasing globally, leading to the expected new strategy of “stent-less PCI." This study aimed to evaluate the one-year outcome of DCB compared to DES.

Methods: Patients who underwent initial PCI for de novo lesions in our institution from January 2018 to December 2021 (n=337) were subjected to retrospective analysis. Among them, 75 patients were treated with DCB, while 262 patients were treated with DES. Target lesion failure (TLF) was evaluated during the follow-up period.

Results: The proportion of PCIs for ACS was significantly lower in the DCB group (DCB, n=23, 30.7% vs. DES, n=143, 54.6%; p=0.001). The median device diameter and length in the DES group were larger than those in the DCB group (DCB, 2.60 mm vs. DES, 2.98 mm; p<0.001; DCB, 19.1 mm vs. DES, 25.2 mm; p<0.001). There was no significant difference between the DCB and DES groups in lesion calcification. The proportion of ostial lesions was significantly higher in the DCB group (DCB, n=13, 17.3% vs. DES, n=21, 8.0%; p=0.018). The cumulative rate of TLF (DCB, n=5, 6.7% vs. DES, n=18, 6.9%; p=0.951) did not significantly differ between the DCB and DES groups.

Conclusion: DCB may be as effective a strategy as DES in the patient who underwent initial PCI for a de novo lesion.

## Introduction

The development of drug-eluting stent (DES) implantation has greatly reduced the risk of restenosis and stent thrombosis and has become the current golden standard in percutaneous coronary intervention (PCI), guaranteeing initial and long-term outcomes [1]. However, long-term outcomes of DES may not be suboptimal in complex lesions such as bifurcation lesions or severely calcified lesions, and DES may be difficult to treat in patients with metal allergies or those who cannot take antiplatelet drugs due to high bleeding risk [2-5]. On the other hand, a drug-coated balloon (DCB) is a non-implantable balloon that applies an antiproliferative drug to the vessel wall without the use of a permanent implant [6]. Currently, in Japan, SeQuent® Please (B. Braun, Germany) has been mainly used, with the addition of Agent™ (Boston Scientific, USA) from 2023, for stent restenosis and coronary angioplasty for small vessels smaller than 3 mm in diameter [7]. Recently, the efficacy of DCB has been reported in patients with acute coronary syndrome and de novo lesions larger than 3.0 mm in diameter [8,9]. Clearly, the number and proportion of DCB utilization within the J-PCI registry have increased in Japan in the last few years. It is assumed that it is due to the accumulation of experience in use and confirmation of efficacy in real-world clinical practice [10]. However, the efficacy of DCB compared to DES in various patients and lesions remains unclear in actual clinical practice. Consequently, we investigated the background of patients treated with DCB or DES and evaluated one-year outcomes.

## Materials and methods

Study population

**Figure 1:**
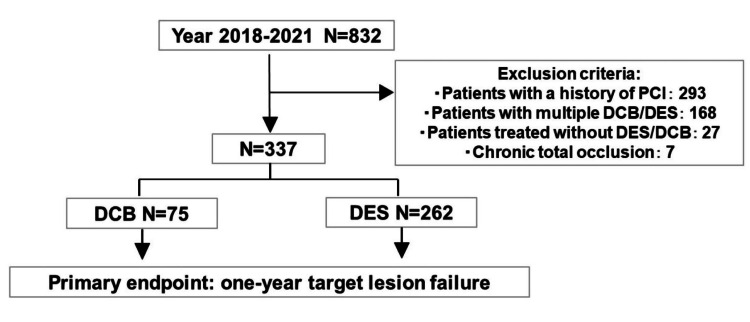
The flowchart of this study

This retrospective study investigated 832 patients who underwent initial PCI for de novo lesions in the Department of Cardiology, Okitama Public General Hospital, Yamagata, Japan, from January 2018 to December 2021. Figure 1 shows the study flowchart. The exclusion criteria were: (1) patients with a history of PCI; (2) patients with multiple DCB/DES; (3) patients treated without DES/DCB; and (4) chronic total occlusion. We investigated 337 patients in this study. The number of patients treated with DCB was 75, and that with DES was 262. The criteria for completion of DCB treatment were the absence of flow-limiting dissection and TIMI (thrombolysis in myocardial infarction) flow grade 3 after PCI. The primary end point was target-lesion failure at one year after the procedure, defined as cardiovascular death, target vessel myocardial infarction, or target lesion revascularization. All procedures were performed in accordance with the principles of the Declaration of Helsinki.

Analysis parameters

Patient information was collected, including age, sex, body mass index (BMI), history of hypertension, dyslipidemia, diabetes, smoking, atrial fibrillation, hemodialysis, estimated glomerular filtration rate (eGFR), left ventricular ejection function (LVEF), brain natriuretic peptide (BNP), and acute coronary syndrome (ACS). Due to ambiguity in assessing lesion length, device length was assessed with reference to the previous study [11]. All patients’ outcomes were confirmed by medical records one year after PCI. The primary endpoint was target lesion failure (TLF), including cardiovascular death, target vessel myocardial infarction, and target lesion revascularization.

Statistical analysis

JMP version 13.2 (SAS Institute Inc., Cary, NC, USA) was used for statistical analysis. Categorical variables were analyzed by the chi-squared test. Quantitative data that conformed to the normal distribution were analyzed by a t-test and expressed as mean ± standard deviation (SD); those that did not conform to the normal distribution were analyzed by a Wilcoxon rank-sum test and expressed as median and quartile range. A Kaplan-Meier curve was used to analyze time-related events. Statistical significance was set at p < 0.05.

## Results

Patient characteristics

The clinical characteristics of the study population are summarized in Table 1.

**Table 1:** Patient characteristics BMI: body mass index; AF: atrial fibrillation; HD: hemodialysis, eGFR: estimated glomerular filtration rate; LVEF: left ventricular ejection function; BNP: brain natriuretic peptide; ACS: acute coronary syndrome Data are expressed as mean ± standard deviation, n (%), or median (inter-quartile range).

	DCB (N=75)	DES (N=262)	p-value
Age, years	72.5 ± 11.3	72.6 ± 11.4	0.908
Female, n (%)	18 (24.0)	67 (25.6)	0.782
BMI, kg/m²	23.1 ± 5.1	24.0 ± 3.3	0.079
Hypertension, n (%)	60 (80.0)	214 (81.2)	0.695
Dyslipidemia, n (%)	54 (72.0)	175 (67.1)	0.417
Diabetes, n (%)	29 (38.7)	102 (39.1)	0.948
Smoke, n (%)	11 (14.7)	53 (20.3)	0.331
AF n (%)	12 (16.0)	47 (18.1)	0.677
HD, n (%)	5 (6.7)	8 (3.1)	0.154
eGFR, mL/min/1.73m²	59.8 ± 21.0	61.2 ± 20.1	0.432
LVEF, %	62.0 ± 12.2	60.8 ± 12.1	0.445
BNP, pg/mL	44.2 (16.3-130.0)	63.2 (22.6-202.0)	0.053
ACS, n (%)	23 (30.7)	143 (54.6)	0.001

A total of 337 patients were included in this study, with 75 treated with DCB and 262 treated with DES. A proportion of ACS in the DCB group was lower than in the DES group (n=23, 30.7% vs. n=143, 54.6%). There were no statistically significant differences between the DCB and DES groups in terms of clinical characteristics, with the exception of ACS.

Lesion and procedural characteristics

The target vessel locations are shown in Table 2.

**Table 2:** Lesion and procedural characteristics LMT: left main trunk; LAD: left anterior descending artery; LCX: left circumflex artery; RCA: right coronary artery; OAS: Orbital atherectomy system; DCA: Directional coronary atherectomy Data are expressed as mean ± standard deviation, n (%), or median (inter-quartile range).

	DCB (N=75)	DES (N=262)	p-value
Target lesion			
LMT, n (%)	4 (5.3)	10 (3.8)	
LAD, n (%)	40 (53.3)	113 (43.1)	
LCX, n (%)	18 (24.0)	48 (18.3)	
RCA, n (%)	13 (17.3)	91 (34.7)	0.040
Multivessel disease	15 (20.0)	104 (39.7)	0.002
Device diameter, mm	2.60 ± 0.55	2.98 ± 0.46	<0.001
Diameter ≥ 3.0 mm, n (%)	30 (40.0)	163 (62.2)	0.001
Device length, mm	19.1 ± 4.0	25.2 ± 9.0	<0.001
Cutting or scoring balloon, n (%)	50 (66.7)	191 (73.2)	0.270
Debulking device			
Rotablator, n (%)	1 (1.3)	0	0.061
OAS, n (%)	8 (10.7)	4 (1.5)	<0.001
DCA, n (%)	16 (21.3)	2 (0.8)	<0.001
Lesion characteristics			
Angiographic calcification	19 (25.3)	83 (31.7)	0.292
>180-degree calcification with imaging	20 (26.7)	75 (28.6)	0.740
Bifurcation lesion	29 (38.7)	92 (35.3)	0.587
Ostial lesion	13 (17.3)	21 (8.0)	0.018
Bendig > 45-degree	8 (10.7)	65 (24.8)	0.009

The left main trunk (LMT) and left anterior descending artery (LAD) tended to be treated more frequently in the DCB group. A proportion of multivessel vessel disease in the DCB group was lower in comparison to the DES group (n=15, 20.0% vs. n=104, 39.7%). The median device diameter and length in the DES group were larger in comparison to the DCB group. There was no significant difference in the utilization of cutting or scoring balloons. The utilization of debulking devices, including rotablators, orbital atherectomy systems (OAS), and directional coronary atherectomy (DCA), in the DCB group was significantly more frequent than that in the DES group. The treatment of ostial lesions was more frequent in the DCB group in comparison to the DES group.

Follow-up TLF

**Figure 2:**
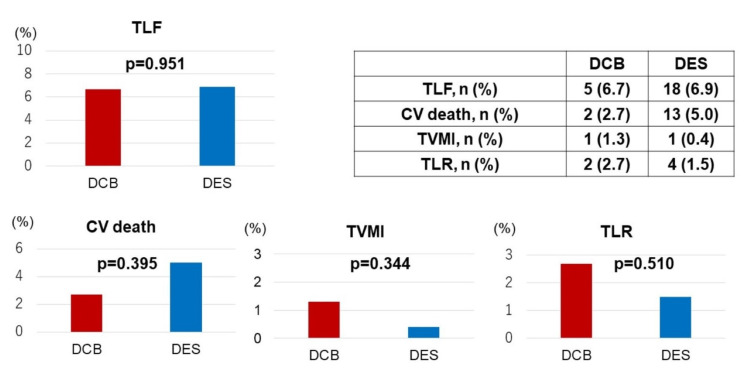
Event ratio of DCB and DES groups DCB: drug-coated balloon; DES: drug-eluting stent; TLF: target lesion failure; CV death: cardiovascular death; TVMI: target vessel myocardial infarction; TLR: target lesion revascularization

Clinical follow-up was available for 294 patients (87.2%). During the follow-up period, there were no significant differences in cardiovascular death, target vessel myocardial infarction, or target lesion revascularization (Figure 2).

**Figure 3:**
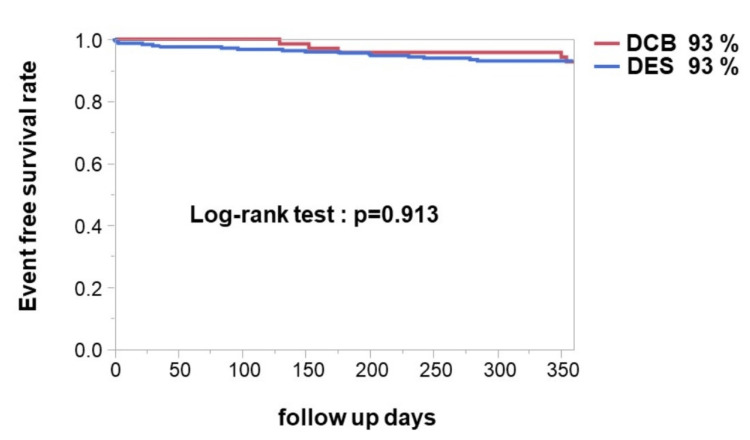
The event-free survival curve of the DCB and DES groups DCB: drug-coated balloon; DES: drug-eluting stent

And the Kaplan-Meyer analysis showed that there were no significant differences in event-free survival between the two groups (Figure 3).

## Discussion

In this study, our findings are as follows: (1) The proportion of ACS and multivessel disease was lower in the DCB group in comparison to the DES group; (2) Kaplan-Meyer analysis revealed that there were no significant differences in event-free survival between the DCB and DES groups; and (3) the utilization of debulking devices in the DCB group was significantly more frequent in comparison to the DES group.

The DES is an established device in terms of safety and efficacy. In this study, one-year TLF in the DES group was similar to previous reports [12]. The outcome of DCB, including patients with acute coronary syndrome and de novo lesions larger than 3.0 mm, was comparable to that of DES in this study. However, the median device diameter and length were larger, and the proportion of ACS and multivessel disease were higher in the DES group in comparison to the DCB group. Therefore, we need to consider that the patient characteristics are not a perfectly matched comparison. Increasing the number of patients to analyze and conduct propensity score matching may eliminate these concerns. The combination of DCA and DCB for large vessel bifurcation lesions is an effective treatment modality with good clinical outcomes [13]. In this study, the utilization of debulking devices, especially DCA, and the treatment of ostial lesions were more frequent in the DCB group (Table 2). Although the study was in patients with stent restenosis, pre-dilation with a scoring balloon before DCB significantly reduced the incidence of restenosis [14]. There was no significant difference in the utilization of cutting or scoring balloons in this study (n=50, 66.7% vs. n=191, 73.2%). However, cutting or scoring balloons were not utilized in 14 patients with DCA in the DCB group. Consequently, the ratio of lesion preparation with cutting or scoring balloons before DCB was considered relatively higher in the DCB group than in the DES group. Optimal lesion preparation is reported to be important in the treatment of DCB [6]. These are listed: 1) a fully inflated balloon of the correct size for the vessel; 2) #30％ residual stenosis; 3) TIMI (thrombolysis in myocardial infarction) flow grade 3; and 4) the absence of a flow-limiting dissection as an indicator that treatment can be completed with DCB. The utilization of debulking devices and cutting balloons is recommended when necessary for this purpose. These factors resulted in a good outcome in the DCB group. And there were no significant differences in the incidence of cardiovascular death, target vessel myocardial infarction, or target lesion revascularization between the two groups during follow-up, also suggesting that DCB is non-inferior to DES in one-year outcomes.

Limitation

Our study has several limitations. Primarily, because it is a retrospective and single-center study, there is the potential for patient selection bias. Second, some lesions, such as long lesions and lesions treated with multiple DCB/DES, were excluded. Therefore, this study would be an evaluation of a de-novo lesion that was treated with a single DCB/DES. Third, the utilization of debulking devices is markedly higher in the DES group, which itself may represent a treatment bias.

## Conclusions

In summary, there was no significant difference in TLF at one year between the DCB and DES groups in patients who underwent initial PCI for de novo lesions, despite some differences in patient backgrounds. The utilization of debulking devices in the DCB group was significantly more frequent in comparison to the DES group. Optimal lesion preparation preceding the utilization of DCB was considered important for a favorable outcome. However, an adequately powered study should be conducted to confirm these preliminary findings.
